# Synovial calprotectin: A potentially useful biomarker for the diagnosis of *Kingella kingae* native arthritis

**DOI:** 10.1002/ccr3.7106

**Published:** 2023-03-24

**Authors:** Anne‐Gallë Leroy, Pascale Bémer, Cyrille Decante, Louise Ruffier d’Epenoux, Eve Tessier, Vincent Crenn, Aurélie Guillouzouic, Stéphane Corvec, The CRIOGO Microbiology Study Group, Didier Tandé, Anne Gougeon, Vincent Cattoir, Chloé Plouzeau, Carole Lemarié, Racheal Chenouard, Marie‐Frédérique Lartigue

**Affiliations:** ^1^ Service de Bactériologie et des Contrôles Microbiologiques CHU Nantes, Nantes Université Nantes France; ^2^ Laboratoire EA 3826 « Thérapeutiques cliniques et expérimentales des infections », IRS2‐Nantes Biotech Nantes Université Nantes France; ^3^ Nantes study group member of CRIOGO (Centre de Référence des Infections Ostéo‐articulaires du Grand Ouest) Nantes France; ^4^ CHU Nantes, Service de Chirurgie Orthopédique Pédiatrique Nantes Université Nantes France; ^5^ Université de Nantes, CHU Nantes Nantes France; ^6^ CHU Nantes, CCOT, service d'orthopédie Nantes Université Nantes France; ^7^ ESGIAI (ESCMID Study Group for Implant‐Associated Infections) member Nantes France

**Keywords:** calprotectin, *Kingella kingae*, septic native arthritis

## Abstract

*Kingella kingae* is a bacteria involved in developing arthritis in children. Its diagnosis remains difficult. We report a case for which a new biomarker, calprotectin measured in the synovial fluid, was strongly positive and a specific molecular test was the only way to diagnose it specifically.

## Abstract 2

*Kingella kingae* remains as a common etiology of child arthritis. Its growth is sometime difficult. Even if synovial fluid (SF) culture in blood bottle or SF‐specific *K. kingae* PCR have improved the diagnosis, a new biomarker such as calprotectin may be useful for the diagnosis.

## 1 INTRODUCTION

Acute osteoarticular infections in children represent medical and surgical emergencies with potentially severe functional sequelae.^1^ Hence, these infections require a rapid and sensitive diagnosis. Molecular diagnostic tools have dramatically improved the diagnostic yield of pediatric septic arthritis (SA). However, more than 20% of these infections remain unconfirmed because no microorganism is identified.^2^ In such situations, the use of inflammatory markers may be helpful to quickly confirm the diagnosis. Serum C‐reactive protein (CRP) level could be a useful tool, even if it is commonly lower during *Kingella kingae* infections–the leading causative agent in young children's arthritis.^2^ Here we report a case of *K. kingae* knee arthritis with a high level of synovial calprotectin, an inflammatory biomarker recently successfully assessed in prosthetic joint infection (PJI).^3^, ^4^

## 2 CASE REPORT

An otherwise healthy 14‐month‐old child was admitted to the pediatric emergency department after a 2‐day history of fever and right‐knee pain leading to lameness. Physical examination revealed an irritable joint with a limited range of motion and swelling. Ultrasound pointed out a joint effusion. Laboratory results showed an increase in serum CRP value (38.6 mg/L, reference range 0–5 mg/L), whereas a complete blood cell count was within normal limits. Knee SA was therefore suspected.

**FIGURE 1:**
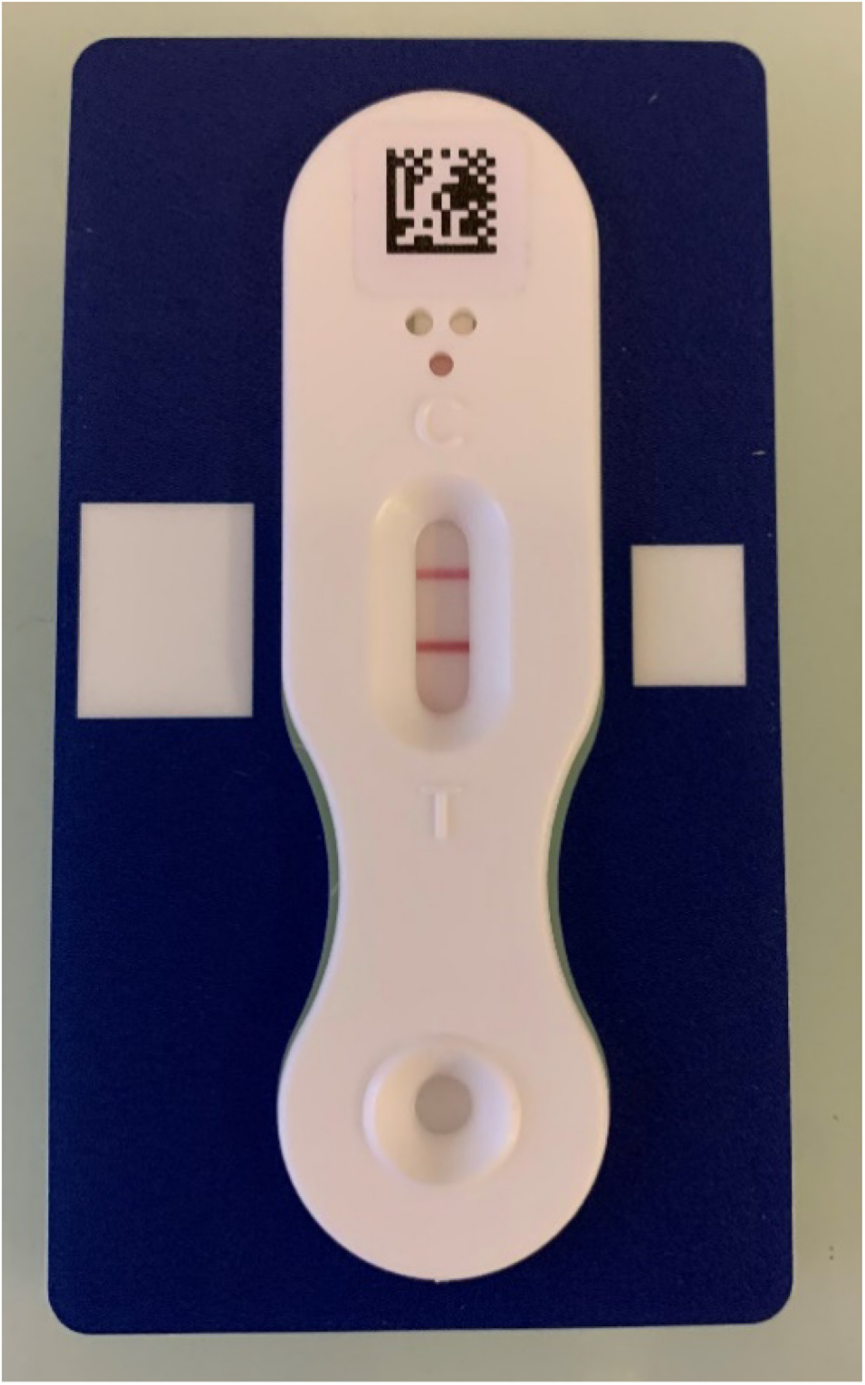
Lyfstone calprotectin test (Lyfstone).

A joint aspiration and an intensive washing with a saline solution were performed. Amoxicillin and clavulanic acid (150 mg per kg of body weight per day) was injected intravenously immediately after surgery. Joint aspiration yielded 5 mL of a purulent fluid sent for cytological and microbiological analysis. A pediatric blood culture vial (Peds Plus/F Becton Dickinson) was promptly inoculated with synovial fluid (SF) in the operating room. White blood cell count showed 142,080 cells/mm^3^ with 91% neutrophils. Unfortunately, Gram staining did not reveal any microorganisms. The joint fluid was inoculated on blood and Polyvitex chocolate agar plates (bioMérieux) incubated for 7 days at 37°C in 5% CO_2_. SF CRP level was measured at 11.7 mg/L. Interestingly, we had an opportunity to assess the SF calprotectin level (a biomarker released from neutrophils upon their activation) which was increased, higher than 300 mg/L (Lysftone), as shown in Figure 1. At that stage, as preliminary laboratory results were in accordance with the SA suspicion, antibiotics were continued.

Despite an optimized culture protocol, every microbiological culture remained sterile after 7 days of incubation. DNA extraction and 16S rRNA gene amplification with universal primers 27f and 1492r were performed directly from the fluid, as previously described.^5^ Despite a 16S rRNA amplification, 16S rRNA gene sequencing failed. Given the age of the patient, a new PCR assay selectively targeting the nonribosomal gene *cpn60* from *K. kingae* was performed and came back positive, confirming the diagnosis.^6^

The clinical outcome was promptly favorable, and the serum CRP level decreased within normal limits 3 days after surgery. Considering recommendations for bone and joint infections in children of her age,^1^ the antibiotic was switched to oral route (amoxicillin/clavulanic acid 500 mg three times per day) and the child was discharged to home on postoperative Day 3. Antibiotic therapy was discontinued 4 weeks after the surgery. At that time, clinical examination and plain radiographs were strictly normal. A favorable outcome without sequelae or any relapse was observed after a 3‐month follow‐up period.

## 3 DISCUSSION

*Kingella kingae*, a Gram‐negative bacterium belonging to the HACEK group, is the leading cause of pediatric osteoarticular infections (mostly SA) in children younger than 4 years.^1^, ^2^ The available published experience on *K. kingae* arthritis pointed out that isolation of this bacterium is challenging. The use of an automated blood culture system enhances the recovery of *K. kingae* from joint fluids.^7^ Most recent studies have demonstrated the obvious superiority of molecular methods, in particular PCR targeting *K. kingae‐*specific genes (*cpn60* or *rtxA* genes), that of *K. kingae* arthritis diagnosis.^6^ In total, these results convincingly demonstrate that (i) the isolation of *K. kingae* remains complicated, even when specimens are seeded into blood culture vials; and that (ii) molecular diagnostic tools increase the detection rate. However, despite the use of molecular methods, more than 20% of pediatric osteoarticular infections persist unconfirmed because no microorganism is detected.^2^

Therefore, other simple tools are needed for acute painful joint diagnosis. Among serum inflammatory markers, procalcitonin shows better diagnosis performances than serum CRP and appears to be the most accurate biomarker in SA, both in adult and pediatric populations. However, due to a serious lack of sensitivity, procalcitonin is not suitable for excluding the diagnosis of SA.^8^ Synovial CRP has been suggested as a useful parameter for the diagnosis of PJI in adults with a relevant cutoff around 10 mg/L, and has even been included in international scoring systems.^9^ Unfortunately, no data are available regarding SF CRP among pediatric SA. In our case, SF CRP was barely above the cutoff.

On the contrary, synovial calprotectin, an important proinflammatory factor of innate immunity released from activated granulocytes, was very high. Recently, synovial calprotectin potential value in the diagnosis of PJI has been stressed.^3^, ^4^ A cutoff of 50 mg/L has been suggested as adequate to diagnose a PJI. Indeed, this fast (15 min), quantitative, and easy to do and read test (by using a smartphone application) can be performed simply in a routine lab. Regarding native joints, few studies recently highlighted that synovial calprotectin could be a relevant biomarker to discriminate SA from other inflammatory arthritis. This new biomarker may help clinicians to decide whether antibiotic therapy is needed or not in conjunction with clinical, radiological, and microbiological findings.^10^

To conclude, we report a case of native SA due to *K. kingae* in a 14‐month‐old child. Despite an optimized culture protocol including a blood culture vial, the negative culture could have led to a diagnostic wandering. The availability (in just a few minutes) of a synovial calprotectin new test highlights its potential usefulness to distinguish juvenile idiopathic arthritis from native joint arthritis. Therefore, this biomarker should be assessed prospectively in this context.

## AUTHOR CONTRIBUTIONS

**Anne‐Gaëlle LEROY:** Conceptualization; formal analysis; resources; validation; writing – original draft; writing – review and editing. **Pascale BEMER:** Conceptualization; formal analysis; resources; writing – original draft; writing – review and editing. **Cyrille DECANTE:** Funding acquisition; resources; writing – review and editing. **Louise RUFFIER D'EPENOUX:** Writing – review and editing. **Eve TESSIER:** Writing – review and editing. **Vincent CRENN:** Writing – review and editing. **Aurélie GUILLOUZOUIC:** Writing – review and editing **Stéphane CORVEC**: Conceptualization; formal analysis; resources; validation; writing – original draft; writing – review and editing.

## FUNDING INFORMATION

Lyfstone (TromsØ, Norway) provided the calprotectin test.

## CONFLICT OF INTEREST STATEMENT

The authors declare no conflict of interest.

## ETHICS STATEMENT

The patient's parents were informed that data from the case would be submitted for publication and gave their consent.

## CONSENT FOR PUBLICATION

Written informed consent was obtained from the patient's parents to publish this report in accordance with the journal's patient consent policy.

## Data Availability

No data sets were used in this article.

## Appendix

CRIOGO Microbiology Study Group: Didier Tandé (CHU Brest), Anne Gougeon, Vincent Cattoir (CHU Rennes), Chloé Plouzeau (CHU Poitiers), Carole Lemarié, Rachel Chenouard (CHU Angers), Marie‐Frédérique Lartigue (CHU Tours).

## References

[1] Lorrot M , Gillet Y , Gras Le Guen C , Launay E , Cohen R , Grimprel E . Antibiotic therapy of bone and joint infections in children: proposals of the French pediatric infectious disease group. Arch Pédiatrie Organe off Sociéte Fr Pédiatrie. 2017;24:S36‐S41. 10.1016/S0929-693X(17)30517-1 29290233

[2] Juchler C , Spyropoulou V , Wagner N , et al. The contemporary bacteriologic epidemiology of osteoarticular infections in children in Switzerland. J Pediatr. 2018;194:190‐196.e1. 29263015 10.1016/j.jpeds.2017.11.025

[3] Wouthuyzen‐Bakker M , Ploegmakers JJW , Ottink K , et al. Synovial calprotectin: an inexpensive biomarker to exclude a chronic prosthetic joint infection. J Arthroplasty. 2018;33:1149‐1153. 29224989 10.1016/j.arth.2017.11.006

[4] Salari P , Grassi M , Barbara C , Onori N , Gigante A . Synovial fluid calprotectin for the preoperative diagnosis of chronic periprosthetic joint infection. J Arthroplasty. 2020;35(2):534‐537. 31542266 10.1016/j.arth.2019.08.052

[5] Aubin GG , Bémer P , Guillouzouic A , et al. First report of a hip prosthetic and joint infection caused by *Lactococcus garvieae* in a woman fishmonger. J Clin Microbiol. 2011;49:2074‐2076. 21367987 10.1128/JCM.00065-11 PMC3122661

[6] Ilharreborde B , Bidet P , Lorrot M , et al. New real‐time PCR‐based method for *Kingella kingae* DNA detection: application to samples collected from 89 children with acute arthritis. J Clin Microbiol. 2009;47:1837‐1841. 19369442 10.1128/JCM.00144-09 PMC2691089

[7] Yagupsky P , Dagan R , Howard CW , Einhorn M , Kassis I , Simu A . High prevalence of *Kingella kingae* in joint fluid from children with septic arthritis revealed by the BACTEC blood culture system. J Clin Microbiol. 1992;30:1278‐1281. 1583131 10.1128/jcm.30.5.1278-1281.1992 PMC265264

[8] Zhao J , Zhang S , Zhang L , et al. Serum procalcitonin levels as a diagnostic marker for septic arthritis: a meta‐analysis. Am J Emerg Med. 2017;35:1166‐1171. 28623003 10.1016/j.ajem.2017.06.014

[9] Parvizi J , Tan TL , Goswami K , et al. The 2018 definition of periprosthetic hip and knee infection: an evidence‐based and validated criteria. J Arthroplasty. 2018;33:1309‐1314.e2. 29551303 10.1016/j.arth.2018.02.078

[10] Couderc M , Peyrode C , Pereira B , et al. Comparison of several biomarkers (MMP‐2, MMP‐9, the MMP‐9 inhibitor TIMP‐1, CTX‐II, calprotectin, and COMP) in the synovial fluid and serum of patients with and without septic arthritis. Joint Bone Spine. 2018;86:261‐262. 29787810 10.1016/j.jbspin.2018.04.008

